# Factors Associated with Practice-Level Performance Indicators in Primary Health Care in Hungary: A Nationwide Cross-Sectional Study

**DOI:** 10.3390/ijerph16173153

**Published:** 2019-08-29

**Authors:** Nóra Kovács, Anita Pálinkás, Valéria Sipos, Attila Nagy, Nouh Harsha, László Kőrösi, Magor Papp, Róza Ádány, Orsolya Varga, János Sándor

**Affiliations:** 1 Department of Preventive Medicine, Faculty of Public Health, University of Debrecen, 4028 Debrecen, Hungary; 2 Department of Financing, National Institute of Health Insurance Fund Management, 1139 Budapest, Hungary; 3 Health Promotion Center, Semmelweis University, 1085 Budapest, Hungary; 4 MTA-DE Public Health Research Group, University of Debrecen, 4028 Debrecen, Hungary; 5 WHO Collaborating Centre on Vulnerability and Health, Department of Preventive Medicine, Faculty of Public Health, University of Debrecen, 4028 Debrecen, Hungary

**Keywords:** general practitioner, primary healthcare, quality indicators, GMP, performance

## Abstract

The performance of general practitioners (GPs) is frequently assessed without considering the factors causing variability among general medical practices (GMPs). Our cross-sectional national-based study was performed in Hungary to evaluate the influence of GMP characteristics on performance indicators. The relationship between patient’s characteristics (age, gender, education) and GMP-specific parameters (practice size, vacancy of GP’s position, settlement type, and county of GMP) and the quality of care was assessed by multilevel logistic regression models. The variations attributable to physicians were small (from 0.77% to 17.95%). The education of patients was associated with 10 performance indicators. Practicing in an urban settlement mostly increased the quality of care for hypertension and diabetes care related performance indicators, while the county was identified as one of the major determinants of variability among GPs’ performance. Only a few indicators were affected by the vacancy and practice size. Thus, the observed variability in performance between GPs partially arose from demographic characteristics and education of patients, settlement type, and regional location of GMPs. Considering the real effect of these factors in evaluation would reflect better the personal performance of GPs.

## 1. Introduction

In many countries, primary healthcare (PHC) systems are performing poorly, causing limited effectiveness of the entire healthcare system [1,2,3]. To meet this challenge, there is a growing trend of applying results-based financing or incentives in PHC systems [4].

The incentives involve the transfer of money or material goods from a funder to a recipient, which is conditional on the recipient taking a measurable action or achieving a predetermined performance target [5]. The evidence is mixed, related to the impact of performance monitoring in PHCs [6,7,8,9]. The negative experiences might be explained by the application of indicators which reflects the influence of factors that cannot be controlled by the GP, instead of the personal performance of the physician [10]. Health care providers are often assessed by the variability in their obtained performance. In this case, the reliable performance indicators have to measure the PHC team’s personal contribution, since the performance can be enhanced by the improvement of the personal contribution. Monitoring based on a set of reliable indicators, which are able to precisely measure the performance of providers, can improve the quality and efficiency of healthcare services and healthcare systems [11,12]. Additionally, upgrading is also important, which ensures their effectiveness through continuous monitoring and refinements [11,13,14]. 

A common organizational and operational unit of general practitioners is the general medical practice (GMP) which provides healthcare services to individuals and families in their communities. The variation in quality of services and health care outcomes across GMPs is partially attributed to the GPs [15,16,17]. GP-independent non-clinical factors, such as patients’ health behavior, psychosocial factors and social circumstances [7,18], health care organizations [16,17], or characteristics of patients (which are also various depending on the geographical location of GMPs) also have an impact on that [15,19,20]. GPs are expected to change those factors over which they have control. On the other hand, the performance monitoring system is expected to measure the GP’s personal performance as specific as it is reasonably possible. Therefore, the evaluation of performance of individual healthcare providers would be more reliable by taking the different patient characteristics and organizational factors into consideration.

Most Central and Eastern European (CEE) countries, including Hungary, offer universal coverage of basic health care services. According to the Primary Health Care Activity Monitor for Europe (PHAMEU) and several studies focusing on the quality of primary care systems [1,3], the overall PHC system performs poorly in Hungary [3], which justifies the application of financial incentives. Although the National Institute of Health Insurance Fund Management (NIHIFM) in Hungary uses process indicators in the assessment of the performance of GPs, the contribution of patients’ and GMP’s characteristics to the variability in indicators has not been assessed yet. Thus, the current evaluation ignores the effects of both socio-demographic and structural composition of GMPs, even though the essential variables are easily available from administrative databases of NIHIFM [21].

The objective of this study was to analyze the variation in performance across GPs with respect to various characteristics of patients (age, gender, education) and GMPs (type of settlement, size of GMP, region of GMP, and vacancy) in an insurance-based health care system.

## 2. Materials and Methods

A cross-sectional study was performed in Hungary to evaluate the influence of GMP characteristics on PHC indicators. This study included all of the GMPs in Hungary that provide PHC for adults. The adult study population was registered in each GMP by gender and age groups of 18–19, 20–24, 25–29, 30–34, 35–39, 40–44, 45–49, 50–54, 55–59, 60–64, 65–69, 70–74, 75–79, 80–84, 85–89, and 90 years and above. Data for the analysis were provided by NIHIFM. This is a secondary analysis of data collected in December 2015. The databases that we analyzed were anonymized. The research protocol was reviewed and permitted by and performed in concordance with the Internal Data Safety and Patient Rights Board of the National Institute of Health Insurance Fund Management. (E01/317-1/2014.)

### 2.1. Setting

The healthcare system in Hungary is based on compulsory health insurance with full coverage. Primary care services are provided by general practitioners (GPs) working in solo practices; therefore, one GMP is operated by one GP whose availability is continuous (filled GP position). By contrast, in vacant GMPs the services are provided by temporary GP with availability restricted in time and place. The GPs are contracted with the NIHIFM, and they receive capitation payments directly from the NIHIFM. The delivery of healthcare services is based on territorial supply obligation, in which municipalities are responsible for providing primary care within their territory, and county governments are responsible for providing specialist healthcare. The 4845 GMPs (in 2015) responsible for adult care are predominantly operating with self-employed GPs organizing the provision on their own authority. There is no market based system with competition between providers; however, patients can choose and change their primary care provider without any restriction [22,23].

### 2.2. Primary Healthcare Indicators

A nationally integrated system of PHC indicators was established by the National Institute of Health Insurance Fund Management (NIHIFM) in Hungary in 2009 (completed with financial incentives in 2010). A set of PHC indicators was developed based on indicators from the Organisation for Economic Co-operation and Development, Healthcare Effectiveness Data and Information Set, and Quality and Outcomes Framework [21]. The legal basis for indicator-based performance assessment of GPs and the rules for evaluating doctors are found in a ministerial decree [24]. Under this rule, the IT network for monitoring was also established, and GPs accepted the monitoring of their performance. This system provides financial incentives for GPs who have reached the desired target values, as defined for regional groups (counties/capital) and the settlement type of GMPs. Approximately 1.7% of the total healthcare funding was dedicated to this purpose in 2010 [25], and was increased gradually up to 4.5% in 2016 [26]. The health policy goals are reflected in defined target values of indicators, but only the most effective 25% of all GMPs meet the required levels [21].The following twelve indicators are used to assess adult PHC by NIHIFM: (1) influenza immunization; (2) mammography screening; (3) proportion of patients who have hypertension among those aged 40–54 years; (4) proportion of patients who have hypertension among those aged 55–69 years; (5) serum creatinine measurement; (6) lipid measurement; (7) beta-blocker application; (8) HbA1c measurement; (9) eye examination; (10) management of chronic obstructive pulmonary disease (COPD); (11) referral rate to secondary care; and (12) antibiotic redemption. Details and definition of assessed process indicators are shown in Table 1. To avoid the repeated counting of performance data, the care events are considered only in the patients’ own general practitioner’s evaluation.

### 2.3. Characteristics of GMPs

All variables in the analyses are out of the control of the physician. Data of patients and GMPs were provided by NIHIFM. The number of patients registered in each GMP was defined by gender and age groups of 18–19, 20–24, 25–29, 30–34, 35–39, 40–44, 45–49, 50–54, 55–59, 60–64, 65–69, 70–74, 75–79, 80–84, 85–89, and 90 years and above.

GMPs were categorized by the number of insured people registered with each GMP (as follows: <800, 801–1200, 1201–1600, 1601–2000, and >2000), types of settlement (rural or urban), and actual status of GMPs (filled or vacant GP position). The regional location of GMPs was described by counties (Baranya, Bács-Kiskun, Békés, Borsod-Abaúj-Zemplén, Csongrád, Fejér, Győr-Moson-Sopron, Hajdú-Bihar, Heves, Komárom-Esztergom, Nógrád, Pest, Somogy, Szabolcs-Szatmár-Bereg, Jász-Nagykun-Szolnok, Tolna, Vas, Veszprém, and Zala counties) and the capital city (Budapest).

The indicator was calculated using gender, age group (7–19, 20–24, 25–29, 30–34, 35–39, 40–44, 45–49, 50–54, 55–59, 60–64, 65–69, 70–74, 75–79, 80–84, 85–89, and 90 years and above), and residence specific data from 2011 Hungarian Census data, which were provided by the Hungarian Central Statistical Office. The summarized length of education was calculated for each place of residence (settlement). The expected number of school years was determined for adults in each settlement by the age and gender composition of the settlement, and the national reference values. The ratio of observed and expected values was used to express the relative education of adults living in their place of residence (settlement-specific relative education) compared with the national reference level. A weighted settlement-specific relative education variable was used by those GMPs, where care was provided for patients from more than one settlement [27].

### 2.4. Statistical Analysis

Regarding binary outcome variables (whether the patients have received care or not) we used multilevel logistic regression models to assess the relationship between patient- and GMP-specific parameters and the performance indicators. The Hosmer-Lemeshow test was used to determine the goodness of fit of the model, which indicated that the model fit the data well. Because of the hierarchical structure of our data, accounting for the clustering of patients within practices was necessary.

Odds ratios (ORs) with the corresponding 95% confidence intervals (CIs) were estimated. The intraclass correlation coefficient (ICC) was presented, which shows the total variance explained by the GP as a grouping factor. 

Statistical significance was considered *p* < 0.05. Statistical analyses were performed using STATA IC version 13.0 software (StataCorp LP., College Station, TX, USA).

## 3. Results

### 3.1. Characteristics of the Studied General Medical Practices

The total number of investigated GMPs that provided PHC for adults was 4845, and the number of registered clients above 18 years was 7,491,888. The proportions of male and female clients were 46.66% (*N* = 3,495,760) and 53.34% (*N* = 3,996,128), respectively.

The majority of GMPs was in urban areas (66.36%, *N* = 3215). Medium-size practices covered the majority of clients; 31.74% of GMPs (*N* = 1538) provided service for 1201–1600 patients and 29.8% of GMPs (*N* = 1444) for 1601–2000 patients. While approximately 20% of GMPs delivered services for more than 2000 patients, 19% of GMPs provided healthcare for a small population (<800 or 801–1200). Furthermore, 4.82% of GMPs were vacant during the study period, as shown in Table 2. 

The proportion of patients receiving care varied between 12.58% (12.56%–12.61%) (referral rate to secondary care) and 77.69% (77.57%–77.81%) (HbA1c measurement) across PHC indicators, as presented in Table 3.

### 3.2. Impact of Potential Influential Factors

The variations attributable to physicians as a grouping factor for process indicators after adjusting for characteristics of patients and practices were small (ICC: from 0.77% to 17.95%). The hypertension care of 55–69-year-old patients (ICC = 0.77%) and 40–54-year-old patients (ICC = 0.86%) had the lowest coefficients. The ICC was lower than 10% for most indicators (beta-blocker application = 2.68%; eye examination = 3.27%; referral rate = 3.35%; serum creatinine measurement = 4.76%; management of COPD = 5%; screening mammography = 5.14%; lipid measurement = 5.35%; antibiotic redemption = 7.83%; HbA1c measurement = 9.16%). The lowest proportion of variation (82.05%) explained by patient factors was observed for influenza immunization (ICC = 17.95%) (Table 4 and Table 5).

According to the results of multilevel analysis, the age and the gender of patients were significant influencing factors of all performance indicators, and the relative education of patients was associated with 10 indicators (excluding beta-blocker application and referral rate). A higher level of education was a positive factor for chronic disease management indicators (management of COPD (OR = 4.17, 3.21–5.42), HbA1c measurement (OR = 3.35, 2.58–4.36), serum creatinine measurement (OR = 2.78, 2.35–3.29), lipid measurement (OR = 2.73, 2.28–3.26), eye examination (OR = 2.22, 1.88–2.63), screening mammography (OR = 2.00, 1.66–2.41) and influenza immunization (OR = 1.69, 1.16–2.46)). The urban settlement type was significantly associated with 9 indicators. Practicing in an urban settlement increased the quality of care mostly for the hypertension and diabetes care related indicators (lipid measurement (OR = 1.25, 1.21–1.29), HbA1c measurement (OR = 1.24, 1.19–1.3) and serum creatinine measurement (OR = 1.23, 1.19–1.27)). The region of GMP was identified as one of the major determinants of variability, but the direction of the effect was not uniform. Using Budapest (the capital city of Hungary) as a reference, significant differences were found among counties. All counties were positively associated with the indicator for hypertension prevalence (among 55–69 year-olds). The vacancy of GMP and size of the GMP significantly affected only a few indicators. There was an unequivocal negative association with influenza immunization, screening mammography, referral rate to secondary care and antibiotic redemption. Patients receiving care from smaller GMPs were less likely to use antibiotics (<800: OR = 0.8, 0.7–0.9; 801–1200: OR = 0.86, 0.81–0.91), while patients of larger practices were more likely to receive mammographies (1601–2000: OR = 1.07, 1.03–1.1; >2000: OR = 1.09, 1.05–1.13) (Table 4 and Table 5).

## 4. Discussion

### 4.1. Main Findings

The observed variability in performance between GPs arose from demographic characteristics of patients, education of patients, and type of settlement and regional localization of GMPs, factors which are beyond the control of the physician. The proportion of total variability attributable to GP after adjusting for characteristics patient and general practice varied between 0.77% and 17.95% across the process indicators. Our findings are similar to those of other studies, which found that low percentage (<20%) of the variance is attributable to the physician for process measures after adjusting for different factors [28,29].

Our results underscored that patient characteristics are contributors to the variation in performance. Education, as a measurable indicator of socio-economic status, was found to be a determinant of the effectiveness of chronic care, including hypertension and diabetes. Such a link between effective hypertension care, diabetes control and the sociodemographic and socio-economic status of patients has been underlined by several papers [20,30,31,32]. 

The settlement type of the GMP was a significant determinant of most indicators in this study. As reported by other articles as well, differences between patterns of acute and preventive services could be observed in rural and urban residences [33,34]. 

Variations in healthcare services were also recognized in different regions of Hungary. These regions should receive extra attention in PHC management, as the fundamental objective of health policy is to reduce health inequities by minimizing unwarranted variations in healthcare services [35].

The GMP size was found to be associated with several indicators (screening mammography, hypertension prevalence, and antibiotic redemption). The finding of a higher screening attendance rate with a larger size of GMP deviates from prior reports [36,37]. The impact of chronic disease management on treatment adherence and patient outcomes remains controversial [37,38,39], and our findings reassure that there is no optimal GMP size to achieve the best healthcare outcomes in general [37,38,39,40,41].

Our results showed that the influence of GMPs’ vacancy on PHC indicators was limited, but the increased rate of vacant GMPs was detected in the study period. A workforce crisis in PHC, as a direct consequence of the aging of GPs, has been observed in many OECD countries [1,42]. Nevertheless, according to PHAMEU, the workforce planning capacity is weak in CEE countries [1]. 

Although the patient’s education, settlement type and regional location of GMP had a considerable effect for most indicators in the study period, the direction of observed effects was variable. Educational level was positively associated with preventive services for chronic diseases (hypertension, diabetes, COPD), influenza immunization and mammography screening. The relationship between the level of education and the use of preventive care services has been established [43,44]. Individuals with a higher educational level are prone to avoid health-risk behaviors and attend preventive healthcare examinations [44]. Moreover, they obtain more preventive care and are more likely to keep their chronic conditions (such as diabetes and hypertension) under control [45]. In the case of settlement type and the county of GMP, which are permanent variables, the variability in the direction of their effects may be caused by frequent changes in legal, funding and organizational environment (e.g., a policy on performance volume limit or patient transport).

### 4.2. Strengths and Limitations

The strength of this study is its use of population-based data, avoiding selection bias in the analysis of the association between characteristics of GMPs and quality of care, which results in the representativeness of our results for the whole population. The continuous data availability allows a regular analysis of different periods without primary data collection. 

A limitation of this study was that the educational level cannot be considered as a year- and GMP-specific indicator. The information on education was not available for the investigated year. Additionally, if more GPs were providing care in one settlement, the same settlement-specific education was considered for each GMP.

Our study is restricted to the existing indicator set available in Hungary, which contains only process indicators for performance assessment. This approach does not allow a comprehensive evaluation of the performance. 

Although our analyses were controlled for age, gender, and education-indicated socio-economic status of patients, and for practice size, vacancy, settlement type and regional location of GMP, there were factors contributing to the presented relationship which could not be incorporated in our models.

A further issue regarding the observed associations is the large sample size, which can detect even small effects with limited importance as statistically significant.

### 4.3. Implication of Findings

To provide an overview of PHC system performance, it is important to collect data on healthcare providers and to evaluate their variability [17]. A good indicator can be used to motivate and encourage doctors to change provision habits. However, if the results from performance assessment systems do not lead to direct benefits for GPs, because patient and GMP characteristics cover the positive effects, there might be a loss of interest by providers in the motivational system. Ignoring the complexity of indicators (additional factors at the patient and GMP level) causes inequitable distribution of available resources, leaving remote areas in need of help without compensation. Adjusted indicators are able to precisely measure the personal performance of the provider and also lead to more effective incentives, and more efficient and equitable use of resources at the GP level. Therefore, understanding the difference between non-adjusted (reflecting the needs of patients regardless of their socio-demographic status, and of the working environment of the GPs) and adjusted (reflecting the GPs’ personal performance) indicator systems is crucial. 

There are suggestions in the literature over standardization of PHC performance indicators for demographic factors, socio-economic and supply conditions [46]. In accordance with this approach, based on the practice of NHS, all Quality and Outcomes Framework payments are weighted by list size and in the clinical domain by disease prevalence [47]. Furthermore, in order to reduce health inequalities in New Zealand via weighting the payment for performance, some indicators are measured individually for “high-need populations”, which are rewarded at a higher rate [48]. In Australia, the practice incentive payments are based on practice patient load (including practice size, age, and gender) and rural loading factors (including geographical size of the region and remoteness of the practice) [48].

Although our findings confirmed that adjustment for patient and GMP characteristics is necessary, further research involving more factors beyond the control of physician is needed to allow for more precise assessment of GPs’ performance. There could be other factors at both patient and GMP levels which may contribute to the variability unexplained in our investigation [20]. To ascertain whether and to what extent the individualization of performance-based incentives for low-performing practices results in an improvement in performance can be a direction for future research. 

## 5. Conclusions

Our study demonstrates that some factors out of the control of GPs (patients’ age, gender, education, settlement type and county of GMP) contribute to the variability in performance across GMPs. As a consequence, the crude performance indicators do not give us information about the personal performance of GP in a comparable way. Taking into consideration that the motivation of GPs can be facilitated by presenting them with their personal performance, elimination of the effect of factors beyond the physician’s control (patient and GMP characteristics) can be a tool for motivation enhancement. 

The poorer the performance of the primary healthcare system in a country, the more important it is to consider the monitoring system based on GP contribution-specific indicators as a resource for PHC development. This kind of indicator could improve the effectiveness of financial incentives as well.

## Figures and Tables

**Table 1:** The list of indicators (with target groups and definitions) used in primary healthcare responsible for the provision to adults in Hungary.

Indicator Name	Target Group	Indicator Definition
Influenza immunization	primary healthcare (PHC) patient over 65 years	Proportion of PHC patients, aged 65 and older, who received an influenza immunization in the previous 12 months
Mammography screening	45–65-year-old female PHC patients	Proportion of female PHC patients, aged 45 to 65, who received mammography in the previous 24 months
Proportion of patients who have hypertension among those aged 40–54 years	40–54-year-old PHC patients	Proportion of patients, aged 40–54 years, who used antihypertensive agents at least 4 times in the previous 12 months
Proportion of patients who have hypertension among those aged 55–69 years	55–69-year-old PHC patients	Proportion of patients, aged 55–69 years, who used antihypertensive agents at least 4 times in the previous 12 months
Serum creatinine measurement	PHC patients with hypertension	Proportion of PHC patients with hypertension screened for serum creatinine in the previous 12 months
Lipid measurement	PHC patients with diabetes and/or hypertension	Proportion of PHC patients with hypertension and/or diabetes screened for lipid abnormalities in the previous 12 months
Beta-blocker application	Patients with myocardial infarction (AMI), or coronary bypass (CABG), or percutaneous transluminal coronary angioplasty (PTCA)	Proportion of patients who used beta-blockers in the previous 12 months
HbA1c measurement	Anatomical Therapeutic Chemical (ATC) A10 drug users	Proportion of PHC patients with diabetes mellitus screened for Haemoglobin A1c (HbA1c) in the previous 12 months
Eye examination	ATC A10 drug users	Proportion of PHC patients with diabetes mellitus who attended eye examination in the previous 12 months
Management of chronic obstructive pulmonary disease (COPD)	ATC R03 drug users and patients with COPD	Proportion of PHC patients with COPD who attended pulmonary function testing in previous 12 months
Referral rate to secondary care	PHC patients	General practitioners (GPs)’ referral rate to secondary care in the previous 6 months
Antibiotic redemption	PHC patients over 18 years	Proportion of redeemed antibiotic prescriptions in the previous 12 months

**Table 2:** Characteristics of general medical practices (GMPs) in 2015.

Characteristics of GMPs	*N* (%)
Age of patients *	49.27
Gender distribution of patients	
Male	3,495,760 (46.66%)
Female	3,996,128 (53.34%)
Relative education of patients **	1.00 (0.1)
Number of GMPs	4845
Number of patients in GMPs	7,491,888
Settlement type of GMP	
rural	1630 (33.64%)
urban	3215 (66.36%)
Panel size (number of patients)	
<800	189 (3.9%)
801–1200	718 (14.82%)
1201–1600	1538 (31.74%)
1601–2000	1444 (29.8%)
>2000	956 (19.73%)
Vacancy	
filled	4611 (95.17%)
vacant ≤ 1 year	85 (1.75%)
vacant 1–4 years	80 (1.65%)
vacant > 4 years	69 (1.42%)

* weighted mean. ** gender- and age-standardised relative education (standard deviation).

**Table 3:** The proportion of patients (with 95% confidence intervals) who received care in 2015 for the whole country by indicators.

Indicators	Number of Patients Received the Care	Number of People in the Target Group	Proportion of Patients Received the Care
Influenza immunization	362,186	1,688,417	21.45% (21.39%–21.51%)
Mammography screening	632,332	1,374,960	45.99% (45.91%–46.07%)
Proportion of patients with hypertension (40–54)	412,385	1,954,786	21.1% (21.04%–21.15%)
Proportion of patients with hypertension (55–69)	1,014,772	1,836,785	55.25% (55.18%–55.32%)
Serum creatinine measurement	1,634,632	2,401,020	68.08% (68.02%–68.14%)
Lipid measurement	1,523,036	2,469,100	61.68% (61.62%–61.74%)
Beta-blocker application	90,564	169,357	53.48% (53.24%–53.71%)
HbA1c measurement	371,857	478,660	77.69% (77.57%–77.81%)
Eye examination	193,542	478,660	40.43% (40.3%–40.57%)
Management of COPD	148,657	191,732	77.53% (77.35%–77.72%)
Referral rate to secondary care	942,821	7,491,804	12.58% (12.56%–12.61%)
Antibiotic redemption	1,765,261	7,491,888	23.56% (23.53%–23.59%)

**Table 4:** Influence of characteristics of patients and general medical practices (GMPs) (Odds Ratio, 95% confidence intervals) on the primary healthcare indicators related to hypertension and diabetes care, according to multilevel logistic regression analysis in Hungary in 2015.

Characteristics of Patients and GMP	Proportion of Patients with Hypertension (40–54)	Proportion of Patients with Hypertension (55–69)	Serum Creatinine Measurement	Lipid Measurement	HbA1c Measurement	Eye Examination
	OR [95%CI]	OR [95%CI]	OR [95%CI]	OR [95%CI]	OR [95%CI]	OR [95%CI]
Gender of patients (Ref.: male)						
female	**1.02 [1.01–1.03]**	**1.16 [1.15–1.17]**	**1.11 [1.1–1.11]**	**1.08 [1.07–1.09]**	**1.02 [1.01–1.04]**	**1.12 [1.11–1.13]**
Age group of patients						
18–19 years	-	-	**0.6 [0.54–0.66]**	**0.78 [0.72–0.85]**	**6.58 [4.73–9.16]**	**0.79 [0.69–0.91]**
20–24 years	-	-	**0.47 [0.45–0.5]**	**0.6 [0.57–0.62]**	**1.79 [1.56–2.07]**	**0.62 [0.56–0.69]**
25–29 years	-	-	**0.46 [0.44–0.48]**	**0.57 [0.55–0.59]**	**1.17 [1.05–1.31]**	**0.67 [0.62–0.73]**
30–34 years	-	-	**0.46 [0.45–0.48]**	**0.54 [0.53–0.56]**	0.97 [0.89–1.06]	**0.7 [0.65–0.75]**
35–39 years	-	-	**0.48 [0.47–0.49]**	**0.54 [0.53–0.55]**	1.03 [0.97–1.1]	**0.66 [0.62–0.69]**
40–44 years	**0.29 [0.28–0.29]**	-	**0.51 [0.5–0.51]**	**0.56 [0.56–0.57]**	**1.09 [1.04–1.15]**	**0.69 [0.66–0.72]**
45–49 years	**0.56 [0.55–0.56]**	-	**0.54 [0.54–0.55]**	**0.6 [0.59–0.61]**	**1.07 [1.03–1.11]**	**0.73 [0.7–0.75]**
50–54 years	Reference	-	**0.64 [0.63–0.64]**	**0.69 [0.68–0.7]**	**1.1 [1.06–1.14]**	**0.78 [0.75–0.8]**
55–59 years	-	**0.41 [0.4–0.41]**	**0.76 [0.75–0.77]**	**0.81 [0.8–0.81]**	**1.08 [1.05–1.11]**	**0.85 [0.83–0.87]**
60–64 years	-	**0.63 [0.62–0.63]**	**0.85 [0.84–0.86]**	**0.88 [0.87–0.89]**	**1.02 [1–1.05]**	**0.89 [0.87–0.91]**
65–69 years	-	Reference	Reference	Reference	Reference	Reference
70–74 years	-	-	**1.08 [1.07–1.09]**	**1.03 [1.02–1.04]**	**0.89 [0.86–0.91]**	**1.05 [1.03–1.07]**
75–79 years	-	-	**1.06 [1.04–1.07]**	**0.95 [0.94–0.96]**	**0.73 [0.71–0.74]**	**0.94 [0.92–0.96]**
80–84 years	-	-	**0.88 [0.87–0.89]**	**0.76 [0.75–0.77]**	**0.53 [0.51–0.55]**	**0.74 [0.72–0.76]**
85–89 years	-	-	**0.67 [0.65–0.68]**	**0.56 [0.55–0.57]**	**0.38 [0.37–0.4]**	**0.53 [0.51–0.55]**
>90 years	-	-	**0.45 [0.44–0.46]**	**0.36 [0.35–0.37]**	**0.25 [0.24–0.27]**	**0.34 [0.32–0.37]**
Relative education of patients	**0.21 [0.19–0.24]**	**0.45 [0.41–0.49]**	**2.78 [2.35–3.29]**	**2.73 [2.28–3.26]**	**3.35 [2.58–4.36]**	**2.22 [1.88–2.63]**
Size of practice (Ref.: 1201–1600)						
<800	0.97 [0.92–1.02]	0.98 [0.93–1.02]	1.04 [0.96–1.12]	1.04 [0.96–1.13]	1.04 [0.93–1.18]	1.02 [0.94–1.1]
801–1200	0.98 [0.96–1.00]	**0.97 [0.95–0.99]**	1 [0.96–1.04]	1 [0.96–1.04]	0.98 [0.92–1.04]	1.03 [0.99–1.07]
1601–2000	1.01 [0.99–1.02]	1.01 [0.99–1.03]	1.02 [0.99–1.06]	1.02 [0.99–1.06]	1.02 [0.97–1.07]	1.01 [0.98–1.04]
>2000	**0.98 [0.96–0.99]**	1.00 [0.98–1.02]	0.99 [0.95–1.02]	0.98 [0.95–1.01]	0.97 [0.92–1.02]	0.98 [0.95–1.01]
Vacancy (Ref.: filled)						
vacant ≤ 1 year	0.98 [0.92–1.03]	**0.94 [0.89–0.99]**	**0.89 [0.82–0.97]**	**0.9 [0.83–0.98]**	**0.87 [0.77–0.99]**	0.99 [0.89–1.1]
vacant 1–4 years	1.04 [0.99–1.11]	0.99 [0.95–1.05]	**0.91 [0.84–0.99]**	0.93 [0.85–1.00]	0.93 [0.81–1.06]	1.01 [0.91–1.13]
vacant > 4 years	1.01 [0.95–1.09]	1.00 [0.94–1.08]	0.92 [0.83–1.01]	0.92 [0.83–1.02]	0.97 [0.83–1.14]	0.98 [0.87–1.1]
Types of settlement (Ref.: rural)						
urban	**0.94 [0.93–0.96]**	**0.97 [0.95–0.98]**	**1.23 [1.19–1.27]**	**1.25 [1.21–1.29]**	**1.24 [1.19–1.3]**	**1.09 [1.06–1.13]**
County of GMP (Ref.: Budapest)						
Baranya	**1.16 [1.13–1.2]**	**1.22 [1.18–1.26]**	1.04 [0.97–1.11]	**0.9 [0.84–0.97]**	**1.4 [1.26–1.56]**	**1.06 [1–1.13]**
Bács-Kiskun	**1.09 [1.06–1.13]**	**1.09 [1.05–1.12]**	**1.1 [1.02–1.18]**	0.96 [0.89–1.03]	**1.32 [1.19–1.47]**	**0.91 [0.85–0.98]**
Békés	**1.09 [1.05–1.14]**	**1.06 [1.02–1.1]**	**0.84 [0.78–0.9]**	**0.79 [0.74–0.85]**	1.06 [0.94–1.2]	**0.75 [0.7–0.8]**
Borsod-Abaúj-Zemplén	**1.26 [1.23–1.3]**	**1.16 [1.13–1.19]**	0.95 [0.9–1]	**0.88 [0.83–0.93]**	**1.15 [1.05–1.25]**	**0.91 [0.86–0.96]**
Csongrád	**1.09 [1.05–1.12]**	**1.11 [1.08–1.15]**	1.02 [0.95–1.1]	0.98 [0.91–1.05]	**1.25 [1.14–1.38]**	1.02 [0.97–1.09]
Fejér	**1.12 [1.08–1.16]**	**1.15 [1.11–1.18]**	0.96 [0.9–1.02]	**0.87 [0.81–0.93]**	**1.13 [1.02–1.25]**	**0.9 [0.85–0.96]**
Győr-Moson-Sopron	**1.1 [1.06–1.14]**	**1.19 [1.15–1.23]**	0.95 [0.88–1.02]	**0.9 [0.83–0.97]**	**1.17 [1.05–1.31]**	**0.67 [0.62–0.72]**
Hajdú-Bihar	**1.1 [1.07–1.14]**	**1.09 [1.06–1.12]**	**1.11 [1.04–1.19]**	0.99 [0.92–1.06]	**1.37 [1.23–1.52]**	1.02 [0.97–1.08]
Heves	**1.21 [1.17–1.26]**	**1.16 [1.12–1.2]**	0.95 [0.89–1.02]	**0.83 [0.77–0.89]**	0.89 [0.8–1]	**0.61 [0.57–0.66]**
Komárom-Esztergom	1.03 [0.99–1.07]	**1.07 [1.04–1.11]**	**0.8 [0.75–0.86]**	**0.73 [0.68–0.78]**	**0.82 [0.74–0.92]**	**0.82 [0.76–0.87]**
Nógrád	**1.16 [1.11–1.21]**	**1.1 [1.06–1.14]**	**0.81 [0.74–0.88]**	**0.76 [0.69–0.83]**	**0.66 [0.57–0.76]**	**0.66 [0.61–0.72]**
Pest	**1.02 [1–1.05]**	**1.06 [1.04–1.09]**	1 [0.95–1.05]	0.98 [0.93–1.03]	0.96 [0.89–1.04]	**0.9 [0.85–0.94]**
Somogy	**1.18 [1.14–1.23]**	**1.27 [1.22–1.31]**	**0.9 [0.82–0.98]**	**0.81 [0.74–0.89]**	0.98 [0.86–1.11]	**0.75 [0.7–0.81]**
Szabolcs-Szatmár-Bereg	**1.2 [1.16–1.25]**	**1.17 [1.14–1.21]**	1.05 [0.98–1.12]	**0.92 [0.86–0.99]**	**1.38 [1.24–1.53]**	**0.91 [0.86–0.96]**
Jász-Nagykun-Szolnok	**1.15 [1.11–1.19]**	**1.06 [1.03–1.1]**	**0.85 [0.79–0.91]**	**0.82 [0.76–0.88]**	1.04 [0.93–1.15]	**0.72 [0.68–0.77]**
Tolna	**1.18 [1.14–1.23]**	**1.23 [1.18–1.28]**	1.02 [0.94–1.1]	0.92 [0.85–1]	**1.2 [1.07–1.34]**	**0.74 [0.69–0.8]**
Vas	**1.17 [1.12–1.22]**	**1.19 [1.14–1.23]**	0.98 [0.89–1.07]	0.91 [0.83–1]	**1.36 [1.17–1.58]**	**0.89 [0.83–0.96]**
Veszprém	**1.07 [1.03–1.1]**	**1.11 [1.07–1.15]**	**0.91 [0.84–0.98]**	**0.79 [0.73–0.86]**	1.04 [0.93–1.16]	1.01 [0.93–1.1]
Zala	**1.12 [1.07–1.16]**	**1.12 [1.08–1.16]**	**0.92 [0.85–0.99]**	**0.89 [0.82–0.96]**	**1.25 [1.11–1.41]**	**0.89 [0.82–0.96]**
ICC	0.86%	0.77%	4.76%	5.35%	9.16%	3.27%

Significant results are shown in bold. ICC: intraclass correlation coefficient.

**Table 5:** Influence of characteristics of patients and general medical practices (GMPs) (Odds Ratio, 95% confidence intervals) on the primary healthcare indicators, according to multilevel logistic regression analysis in Hungary in 2015.

Characteristics of Patients and GMP	Influenza Immunization	Screening Mammography	Beta-blocker application	Management of COPD	Referral rate	Antibiotic redemption
	OR [95%CI]	OR [95%CI]	OR [95%CI]	OR [95%CI]	OR [95%CI]	OR [95%CI]
Gender of patients (ref.: male)						
female	**0.8 [0.79–0.81]**	**-**	**1.14 [1.11–1.16]**	**0.92 [0.9–0.94]**	**1.51 [1.51–1.52]**	**1.69 [1.68–1.7]**
Age group of patients						
18–19 years	-	-	**0.02 [0–0.12]**	**0.67 [0.57–0.79]**	**0.34 [0.33–0.35]**	**1.9 [1.87–1.94]**
20–24 years	-	-	**0.09 [0.05–0.18]**	**0.57 [0.52–0.64]**	**0.27 [0.27–0.28]**	0.99 [0.98–1.01]
25–29 years	-	-	**0.15 [0.1–0.24]**	**0.59 [0.52–0.67]**	**0.28 [0.27–0.28]**	**0.85 [0.84–0.86]**
30–34 years	-	-	**0.29 [0.22–0.38]**	**0.68 [0.6–0.77]**	**0.3 [0.3–0.31]**	**0.89 [0.88–0.90]**
35–39 years	-	-	**0.49 [0.43–0.57]**	**0.81 [0.73–0.89]**	**0.31 [0.3–0.31]**	**0.93 [0.92–0.94]**
40–44 years	-	-	**0.57 [0.52–0.62]**	0.95 [0.88–1.03]	**0.32 [0.32–0.32]**	**0.89 [0.88–0.9]**
45–49 years	-	**0.69 [0.68–0.7]**	**0.74 [0.7–0.79]**	0.98 [0.92–1.04]	**0.41 [0.4–0.41]**	**0.9 [0.89–0.91]**
50–54 years	-	**0.81 [0.8–0.82]**	**0.87 [0.83–0.91]**	**1.12 [1.06–1.18]**	**0.56 [0.55–0.56]**	**0.96 [0.95–0.97]**
55–59 years	-	**0.92 [0.91–0.93]**	**0.92 [0.89–0.96]**	**1.16 [1.11–1.22]**	**0.76 [0.75–0.77]**	**1.04 [1.03–1.05]**
60–64 years	-	Reference	0.99 [0.96–1.03]	**1.05 [1.00–1.09]**	**0.84 [0.83–0.85]**	1.00 [0.99–1.01]
65–69 years	Reference	-	Reference	Reference	Reference	Reference
70–74 years	**1.46 [1.44–1.47]**	-	0.98 [0.95–1.01]	**0.81 [0.78–0.85]**	**1.13 [1.12–1.14]**	0.99 [0.98–1]
75–79 years	**1.86 [1.84–1.89]**	-	**0.93 [0.9–0.96]**	**0.71 [0.68–0.74]**	**1.16 [1.14–1.17]**	0.99 [0.99–1.01]
80–84 years	**2.05 [2.02–2.08]**	-	**0.83 [0.8–0.87]**	**0.47 [0.44–0.49]**	1 [0.99–1.01]	**0.98 [0.97–0.99]**
85–89 years	**1.96 [1.92–2]**	-	**0.76 [0.72–0.8]**	**0.26 [0.25–0.28]**	**0.76 [0.74–0.77]**	**0.97 [0.95–0.99]**
>90 years	**1.78 [1.73–1.83]**	-	**0.67 [0.62–0.73]**	**0.13 [0.11–0.14]**	**0.48 [0.46–0.49]**	**1.04 [1.01–1.06]**
Relative education of patients	**1.69 [1.16–2.46]**	**2.00 [1.66–2.41]**	0.84 [0.68–1.03]	**4.17 [3.21–5.42]**	0.98 [0.84–1.13]	**0.53 [0.42–0.67]**
Size of practice (Ref.: 1201–1600)						
<800	0.96 [0.82–1.13]	0.99 [0.91–1.09]	0.98 [0.88–1.09]	1.12 [0.99–1.27]	0.98 [0.91–1.05]	**0.8 [0.7–0.9]**
801–1200	0.95 [0.88–1.04]	0.99 [0.96–1.04]	1 [0.95–1.05]	1.02 [0.97–1.08]	0.98 [0.95–1.02]	**0.86 [0.81–0.91]**
1601–2000	1.01 [0.95–1.07]	**1.07 [1.03–1.1]**	1 [0.97–1.04]	1.01 [0.97–1.06]	1.01 [0.98–1.03]	1.02 [0.99–1.06]
>2000	1.01 [0.94–1.08]	**1.09 [1.05–1.13]**	1.01 [0.98–1.05]	0.98 [0.94–1.03]	1.01 [0.98–1.04]	**1.07 [1.03–1.12]**
Vacancy (Ref.: filled)						
vacant ≤ 1 year	**0.78 [0.65–0.94]**	**0.77 [0.68–0.87]**	1.04 [0.92–1.18]	0.93 [0.82–1.05]	**0.74 [0.67–0.83]**	**0.26 [0.19–0.36]**
vacant 1–4 years	**0.73 [0.58–0.92]**	**0.85 [0.74–0.98]**	1 [0.87–1.14]	1.06 [0.92–1.21]	**0.73 [0.64–0.83]**	**0.01 [0.01–0.01]**
vacant > 4 years	**0.62 [0.46–0.85]**	**0.82 [0.72–0.93]**	1.05 [0.9–1.23]	0.94 [0.77–1.13]	**0.84 [0.75–0.93]**	**0.01 [0.01–0.01]**
Types of settlement (Ref.: rural)						
urban	0.95 [0.89–1.02]	1.02 [0.98–1.05]	**0.96 [0.92–0.99]**	**1.12 [1.07–1.17]**	**1.07 [1.04–1.1]**	0.98 [0.94–1.02]
County of GMP (Ref.: Budapest)						
Baranya	**1.19 [1.03–1.39]**	**1.17 [1.1–1.25]**	**1.23 [1.15–1.32]**	**1.13 [1.02–1.25]**	**1.21 [1.15–1.27]**	**1.24 [1.12–1.38]**
Bács-Kiskun	1.09 [0.96–1.23]	**1.7 [1.6–1.8]**	1 [0.93–1.07]	**1.32 [1.2–1.45]**	1.01 [0.95–1.07]	**1.58 [1.44–1.73]**
Békés	0.87 [0.75–1]	**1.14 [1.07–1.22]**	**1.27 [1.18–1.37]**	1.1 [0.99–1.21]	**0.94 [0.89–0.99]**	**1.55 [1.4–1.73]**
Borsod-Abaúj-Zemplén	0.99 [0.88–1.1]	**1.61 [1.53–1.69]**	**1.48 [1.38–1.58]**	**0.89 [0.82–0.96]**	**1.37 [1.31–1.43]**	**1.52 [1.41–1.64]**
Csongrád	0.92 [0.82–1.05]	**1.92 [1.81–2.03]**	1.04 [0.97–1.12]	**1.18 [1.08–1.3]**	0.98 [0.93–1.03]	**1.5 [1.38–1.62]**
Fejér	1.01 [0.87–1.18]	**1.4 [1.32–1.49]**	**1.11 [1.04–1.2]**	**1.16 [1.05–1.28]**	**1.09 [1.04–1.14]**	**1.21 [1.12–1.32]**
Győr-Moson-Sopron	**0.71 [0.6–0.83]**	**1.44 [1.36–1.52]**	**1.71 [1.6–1.83]**	**1.23 [1.11–1.36]**	**0.72 [0.69–0.76]**	**1.09 [1.01–1.18]**
Hajdú-Bihar	0.9 [0.8–1.01]	**1.53 [1.44–1.62]**	**1.27 [1.18–1.37]**	**1.39 [1.26–1.53]**	**1.28 [1.22–1.33]**	**1.27 [1.16–1.39]**
Heves	0.88 [0.74–1.03]	1.01 [0.94–1.08]	**1.46 [1.34–1.59]**	**1.31 [1.2–1.43]**	**1.14 [1.09–1.2]**	**1.37 [1.25–1.51]**
Komárom-Esztergom	0.89 [0.71–1.12]	**1.37 [1.27–1.49]**	**0.86 [0.78–0.95]**	**1.56 [1.4–1.75]**	**0.92 [0.86–0.98]**	**1.2 [1.11–1.3]**
Nógrád	1.09 [0.9–1.32]	**0.82 [0.74–0.91]**	1.08 [0.97–1.2]	1.06 [0.95–1.18]	0.98 [0.92–1.05]	**1.34 [1.21–1.49]**
Pest	0.97 [0.87–1.08]	0.94 [0.88–1]	**1.14 [1.08–1.21]**	**1.1 [1.02–1.19]**	**0.95 [0.91–0.99]**	1.03 [0.97–1.1]
Somogy	**1.57 [1.33–1.85]**	**0.69 [0.64–0.74]**	**1.2 [1.11–1.3]**	0.97 [0.88–1.06]	1.03 [0.98–1.09]	**1.23 [1.13–1.34]**
Szabolcs-Szatmár-Bereg	0.98 [0.85–1.12]	**1.63 [1.5–1.77]**	**1.95 [1.82–2.1]**	1.06 [0.98–1.16]	**1.09 [1.03–1.15]**	**1.73 [1.6–1.88]**
Jász-Nagykun-Szolnok	0.89 [0.77–1.04]	**1.51 [1.41–1.61]**	**1.14 [1.05–1.24]**	1.07 [0.94–1.22]	**0.93 [0.88–0.98]**	**1.37 [1.23–1.52]**
Tolna	**1.29 [1.06–1.57]**	**1.75 [1.64–1.87]**	**1.71 [1.55–1.88]**	**0.72 [0.64–0.82]**	**0.47 [0.4–0.56]**	**1.45 [1.31–1.61]**
Vas	**0.83 [0.7–0.99]**	1.01 [0.87–1.18]	**1.69 [1.54–1.85]**	0.98 [0.88–1.09]	**0.88 [0.83–0.93]**	0.97 [0.88–1.08]
Veszprém	**1.31 [1.12–1.52]**	**1.2 [1.12–1.3]**	**1.11 [1.03–1.2]**	**1.9 [1.71–2.1]**	**0.83 [0.78–0.88]**	**1.19 [1.1–1.29]**
Zala	**1.28 [1.12–1.47]**	**1.12 [1.02–1.23]**	0.94 [0.87–1.02]	**1.39 [1.24–1.55]**	**1.11 [1.05–1.18]**	**1.4 [1.27–1.53]**
ICC	17.95%	5.14%	2.68%	5.00%	3.35%	7.83%

Significant results are shown in bold. ICC: intraclass correlation coefficient.
